# Age-dependent dendrobine biosynthesis in *Dendrobium nobile*: insights into endophytic fungal interactions

**DOI:** 10.3389/fmicb.2023.1294402

**Published:** 2023-12-08

**Authors:** Yongxia Zhao, Xiaolong Ji, Xiaoqi Liu, Lin Qin, Daopeng Tan, Di Wu, Chaojun Bai, Jiyong Yang, Jian Xie, Yuqi He

**Affiliations:** ^1^Guizhou Engineering Research Center of Industrial Key-Technology for Dendrobium nobile, Engineering Research Center of Pharmaceutical Orchid Plant Breeding, High Efficiency Application in Guizhou Province, Zunyi Medical University, Zunyi, China; ^2^Key Laboratory of Basic Pharmacology of Ministry of Education, Joint International Research Laboratory of Ethnomedicine of Ministry of Education, Zunyi Medical University, Zunyi, China; ^3^2011 Cooperative Inovational Center for Guizhou Traditional Chinese Medicine and Ethnic Medicine, Zunyi Medical University, Zunyi, China; ^4^Guangxi Shenli Pharmaceutical Co., Ltd., Yulin, China; ^5^Chishui Xintian Chinese Medicine Industry Development Co., Ltd., Zunyi, China

**Keywords:** *Dendrobium nobile*, endophytic fungi, transcriptomic analysis, dendrobine, age-dependent accumulation

## Abstract

**Introduction:**

*Dendrobium nobile (D. nobile)*, a valued Chinese herb known for its diverse pharmacological effects, owes much of its potency to the bioactive compound dendrobine. However, dendrobine content varies significantly with plant age, and the mechanisms governing this variation remain unclear. This study delves into the potential role of endophytic fungi in shaping host-microbe interactions and influencing plant metabolism.

**Methods:**

Using RNA-seq, we examined the transcriptomes of 1-year-old, 2-year-old, and 3-year-old *D. nobile* samples and through a comprehensive analysis of endophytic fungal communities and host gene expression in *D. nobile* stems of varying ages, we aim to identify associations between specific fungal taxa and host genes.

**Results:**

The results revealing 192 differentially expressed host genes. These genes exhibited a gradual decrease in expression levels as the plants aged, mirroring dendrobine content changes. They were enriched in 32 biological pathways, including phagosome, fatty acid degradation, alpha-linolenic acid metabolism, and plant hormone signal transduction. Furthermore, a significant shift in the composition of the fungal community within *D. nobile* stems was observed along the age gradient. *Olipidium*, *Hannaella*, and *Plectospherella* dominated in 1-year-old plants, while *Strelitziana* and *Trichomerium* prevailed in 2-year-old plants. Conversely, 3-year-old plants exhibited additional enrichment of endophytic fungi, including the genus *Rhizopus*. Two gene expression modules (mediumpurple3 and darkorange) correlated significantly with dominant endophytic fungi abundance and dendrobine accumulation. Key genes involved in dendrobine synthesis were found associated with plant hormone synthesis.

**Discussion:**

This study suggests that the interplay between different endophytic fungi and the hormone signaling system in *D. nobile* likely regulates dendrobine biosynthesis, with specific endophytes potentially triggering hormone signaling cascades that ultimately influence dendrobine synthesis.

## 1 Introduction

*Dendrobium nobile* (*D. nobile*) is a precious medicinal herb found in the Danxia landform of Chishui City, Guizhou Province, China. It possesses a rich repertoire of bioactive compounds with notable antioxidant, anti-tumor, and immunomodulatory properties (Teixeira and Ng, 2017). *D. nobile* is considered a rare and valuable resource in Chinese herbal medicine, primarily obtained through wild-simulated cultivation. In its natural habitat, *D. nobile* thrives in clusters on Danxia rocks. Each cluster of *D. nobile* plants produces new stems and leaves every year, while the stems from previous years continue to grow. As a result, a cluster of Dendrobium plants may contain stems of 1-year-old, 2-year-old, 3-year-old and older ages. Interestingly, only the 1-year-old stems have leaves, while the older stems are leafless, and most of the 3-year-old stems produce new shoots at the nodes. Therefore, the age of the Dendrobium stems can be distinguished by these features. The chemical composition and content of Chinese herbs vary with different ages, and *D. nobile* is no exception (Lu et al., 2022).

However, the morphology and chemistry of plants are determined by a combination of gene expression and environmental factors. Environmental factors include not only light, humidity, temperature, etc., but also the microbial community within the plant, which is called endophyte and plays an important role in the plant’s successional trajectory (Clay and Holah, 1999; Afkhami and Strauss, 2016), the balance between insects and plants, plant reproduction, and the accumulation of plant metabolites (Harrison et al., 2021). Interactions between symbiotic or parasitic microorganisms and their host plants are essential for maintaining plant homeostasis (Ma et al., 2021). Research in this area has entered a phase of rapid development, but our understanding of its microbe-hosts is still limited.

The main bioactive constituents of *D. nobile* are alkaloids, with dendrobine being a prominent compound regulated by the Pharmacopeia. Transcriptome analyzes have indicated that dendrobine is derived from the terpenoid-forming and indole pathways of the mevalonate pathway (MVA), the methylerythritol phosphate pathway (MEP), or the shikimate pathway (Wang et al., 2020). However, the details of these biosynthetic pathways to synthesize alkaloids are not well studied. We found that the content of dendrobine was closely related to the age of *D. nobile* and reached a maximum in the youngest 1-year-old plants. Moreover, we observed that the community and abundance of endophytes in 1-year-old *D. nobile* were significantly different from those in older plants. It has also been reported that some endophytic fungi can positively promote the accumulation of fatty acid metabolites in *D. nobile* by infestation and colonization (Zhao et al., 2023). Therefore, we hypothesize that endophyte colonization in 1-year-old *D. nobile* is closely related to the high content of dendrobine alkaloids. By performing transcriptome analysis on *D. nobile* samples of different ages, we identified a number of changes, including changes in some genes associated with the dendrobine synthesis pathway as well as changes in genes in other related pathways.

To validate this hypothesis, we conducted an extensive analysis of the endophytic fungal communities inhabiting *D. nobile* plants of varying ages and investigated their correlation with dendrobine content. Employing high-throughput sequencing technology, we identified and quantified the endophytic fungi residing within the stems of *D. nobile* plants. Subsequently, we performed a comprehensive analysis to determine the relationship between the relative abundance of specific fungal taxa and the levels of dendrobine present. Our findings not only shed new light on the intricate involvement of endophytic fungi in the regulation of dendrobine synthesis and accumulation in *D. nobile* but also hold significant implications for the cultivation and utilization of this invaluable medicinal plant.

## 2 Materials and methods

### 2.1 Sample collection

**Figure 1:**
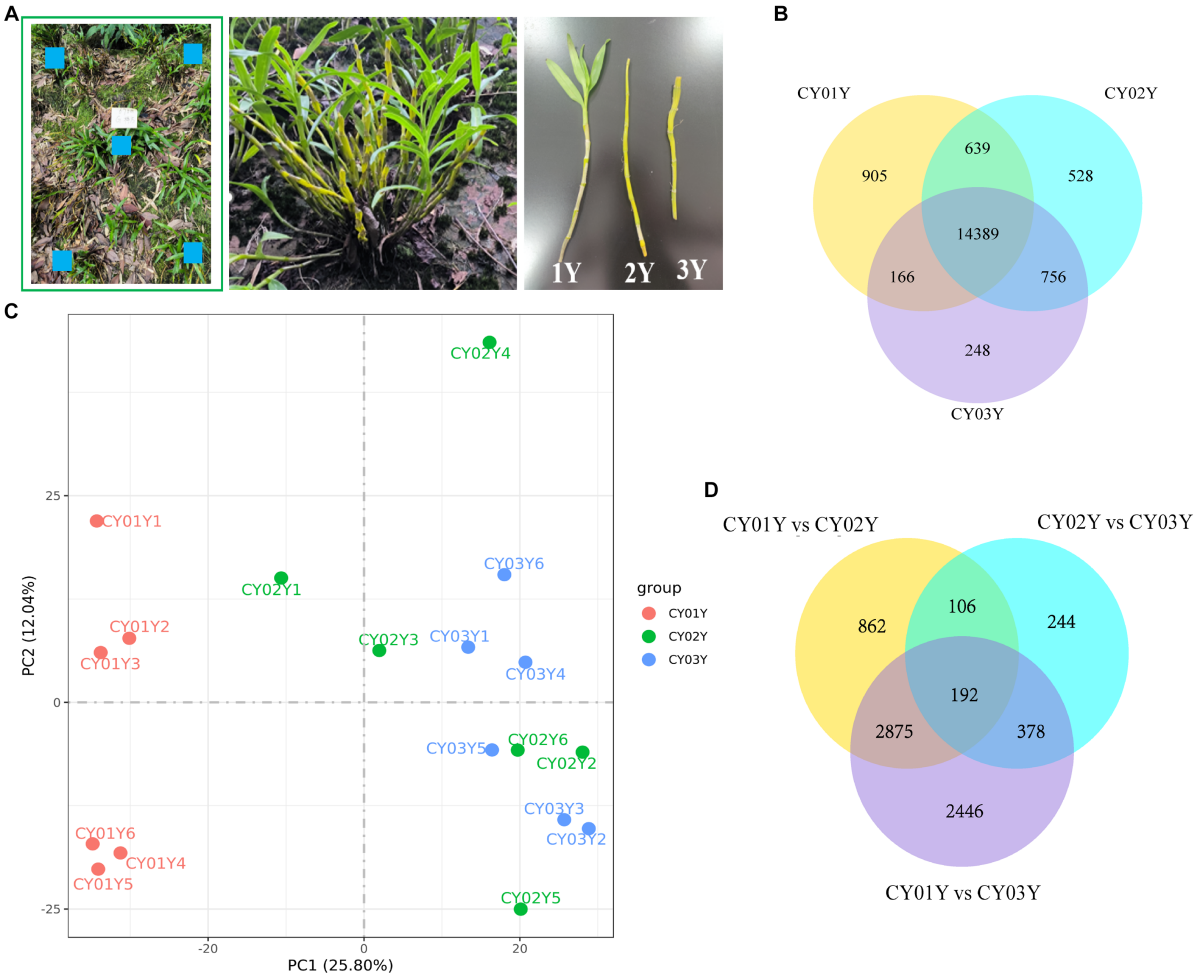
Samples collection and transcriptomic landscape of *D. nobile* stems along the years axis. **(A)** Schematic diagram of sampling, using a five-point systematic sampling method, with samples of one-, two-, and three-year old plants taken from the same cluster. **(B)** Co-expression Venn diagram of *D. nobile* with different ages, showing that there are 14,389 co-expressed genes in the stems of *D. nobile* of different ages. At the same time, there are 905 genes uniquely expressed in 1-year-old plants, 528 genes in 2-year-old plants and 248 genes in 3-year-old plants. **(C)** Principal component analysis (PCA) of the samples, showing that the samples of the CY01Y group are clustered together, while the samples of the CY02Y and CY03Y groups are merged together. Thus, the differences between the CY02Y and CY03Y groups are smaller, while the differences with the CY01Y group are larger. **(D)** Venn diagram for differential expression, showing that differential genes were more abundant between CY01Y and CY02Y as well as CY03Y, whereas the difference between CY02Y and CY03Y was the smallest and the number of differential genes was the least.

*D. nobile* stems of three age groups (1-year-old, 2-year-old and 3-year-old) in six biological replicates were collected from wild-simulated cultivation on Danxia rocks in ChiYan base in Chishui city, Guizhou (N 28°30′2″, E 105°55′48″) by using systematic sampling methods (Figure 1A). After the roots and leaves had been removed, the stems were divided into two parts: one part, containing 200 mg of each sample, was disinfected in the following way. First, the samples were immersed in 20 mL of 75% alcohol for 45 s. Second, after removing the alcohol, the samples were immersed in 20 mL of 0.1% mercuric chloride for 5 min. Finally, the samples were rinsed with sterile water three times to remove the remaining mercuric chloride. All of the stems were then sent to Novogene for the sequencing of the internal transcribed spacer (ITS) with −80°C stored process. The other part of fresh *D. nobile* stems was flash-frozen in liquid nitrogen and transported on dry ice to Novogene for RNA extraction and subsequent experiments.

### 2.2 DNA extraction and sequencing of endophytic fungi

To characterize the endophytic fungal community in *D. nobile* stems of different ages, we employed the CTAB/SDS method for total genomic DNA extraction from each sample. The ITS genes were then amplified in specific regions using designated primers (Forward: GGAAGTAAAAGTCGTAACAAGG; Reverse: GCTGCGTTCTTCA TCGATGC) along with respective barcodes. Subsequently, we conducted library construction and sequencing on an Illumina NovaSeq platform, generating 250 bp paired-end reads following amplification, purification, and library construction as briefly outlined below: Amplification protocol: 98°C (1 min) → (98°C (10 s) → 50°C (30 s) → 72°C (30 s) (30 cycles)) → 72°C (5 min). The PCR products were homogenously combined and then subjected to purification using the Qiagen Gel Extraction Kit (Qiagen, Germany). For library preparation, we employed the NEBNext® Ultra™ II DNA Library Prep Kit (Cat No. E7645). Subsequently, the library’s quality was assessed utilizing the Qubit@ 2.0 Fluorometer (Thermo Scientific) and the Agilent Bioanalyzer 2,100 system.

### 2.3 Transcriptome sequencing

To profile the transcriptomes of 18 stems from *D. nobile* of varying ages, we initiated the process by extracting total RNA from the plant samples. Subsequently, mRNA was isolated from the total RNA pool using poly-T oligo-attached magnetic beads. Prior to this, we employed the RNA Nano 6,000 Assay Kit on the Bioanalyzer 2,100 system to evaluate RNA integrity. Of paramount importance was the initiation of first strand cDNA synthesis, where a combination of random hexamer primers and M-MuLV Reverse Transcriptase (RNase H-) played a crucial role. Following this, DNA Polymerase I and RNase H were enlisted to synthesize the second strand of cDNA. The pivotal step in this process was library construction. Here, the AMPure XP system (Beckman Coulter, Beverly, United States) was utilized to meticulously purify and select cDNA fragments falling within the 370 ~ 420 bp range. Finally, the library preparations were subjected to sequencing on an Illumina Novaseq platform.

### 2.4 Data analysis of ASVs of endophytic fungi

The 18 samples were sequenced on the Illumina NovaSeq platform. We processed the raw reads by removing the barcodes and primer sequences according to barcode sequences. Paired-end reads were merged using FLASH.^1^ To obtain high-quality Clean Tags, we used the fastp (Version 0.20.0) software to filter out low-quality reads. The Vsearch (Version 2.15.0) was used to detect the chimeric sequences, and then the chimeric sequences were removed to obtain the Effective Tags. ASVs Denoise and species annotation was realized in the QIIME2 software (Version QIIME2-202006), and then ASVs with abundance less than 5 were filtered out. The ASVs absolute abundance was normalized using a standard of sequence number corresponding to the sample with the least sequences. Subsequent analysis of alpha diversity and beta diversity were all performed based on the normalized data and calculated in QIIME2. Principal Coordinate Analysis (PCoA) was performed with QIIME2 package, ade4 package and ggplot2 package in R software (Version 3.5.3). To identify the significantly different taxa at each taxonomic level (Phylum, Class, Order, Family, Genus, Species), T-test analysis was performed using R software (Version 3.5.3).

### 2.5 Data analysis of RNA-Seq

Clean data (clean reads) in fastq format was first processed by fastp software to remove adapter, poly-N and low-quality reads. The high-quality clean data was then analyzed for Q20, Q30 and GC content. Hisat2 v2.0.5 and StringTie (v1.3.3b) were adopted to map reads to the reference genome and predict novel transcripts. The number of reads mapped to each gene was counted using Feature Counts v1.5.0-p3. Finally, the FPKM (fragments per kilobase of transcript per million mapped reads) of each gene was calculated. FPKM provides a normalized measure of gene expression, factoring in sequencing depth and gene length, and is widely adopted for estimating gene expression levels. Differential expression analysis was performed between two groups, each consisting of two biological replicates per condition, using the DESeq2 R package (1.20.0). DESeq2 employs statistical routines based on the negative binomial distribution to determine differential expression in digital gene expression data. The resulting *p*-values were adjusted using the Benjamini and Hochberg’s approach to control the false discovery rate. Genes with an adjusted *p*-value of <=0.05, as determined by DESeq2, were identified as differentially expressed. Gene Ontology (GO) enrichment analysis of differentially expressed genes was implemented by the clusterProfiler R package, then to test the statistical enrichment of differentially expressed genes in KEGG pathways.^2^ Eventually, WGCNA (Weighted correlation network analysis) was performed through the R package WGCNA.

## 3 Results

### 3.1 Different transcriptomic landscapes in developmental gradients along the year axis

In this study, we examined the *Dendrobium nobile* stems (Figure 1A) from three distinct age groups: 1-year-old seedlings with leaves (CY01Y), 2-year-old bare stems without leaves (CY02Y), and 3-year-old stems that have sprouted (CY03Y). We conducted both a transcriptomics experiment and an endogenous fungi analysis. Gene expression in the stems of all 18 samples (with six biological replicates per age group) was assessed, and it is noteworthy that all samples exhibited comparable read counts (averaging 42,895,882, 41,514,350, and 44,681,382 clean reads, respectively). Additionally, all samples maintained an average quality score exceeding 30 phred (Supplementary Table S1).

The co-expression Venn diagram (Figure 1B) demonstrated that 905 genes were uniquely expressed in 1-year-old plants, 528 genes in 2-year-old plants, and 248 genes in 3-year-old plants. Moreover, a substantial overlap of 14,389 genes was observed across all three age groups. Principal component analysis (PCA) and differential gene analysis (Figures 1C,D) further illustrated the high degree of transcriptomic similarity between 2- and 3-year-old plants when compared to the 1-year-old plants.

**Figure 2:**
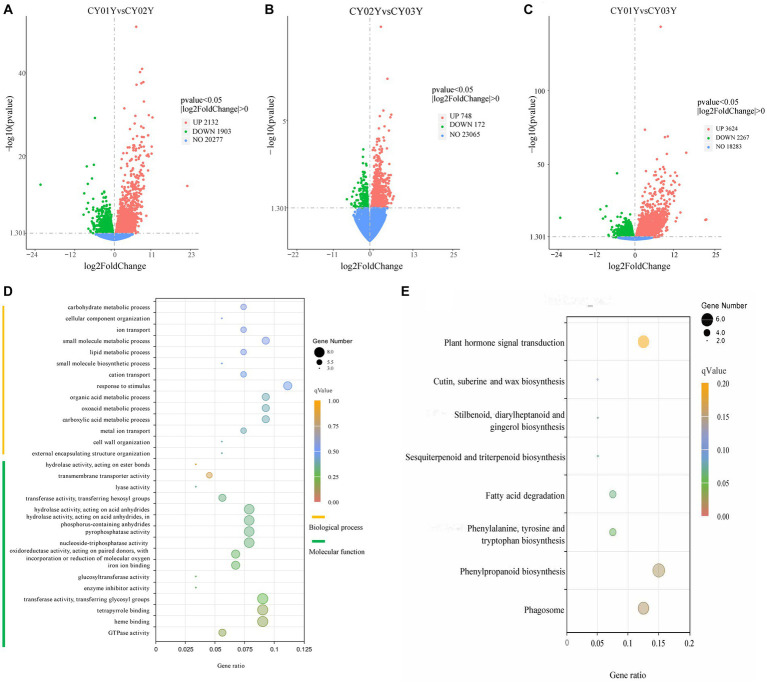
Screening of differentially expressed genes and Gene Ontology (GO) term enrichment analysis and KEGG enrichment. **(A)** The volcano plot for CY01Y and CY02Y comparisons. **(B)** The volcano plot for CY02Y and CY03Y comparisons. **(C)** The volcano plot for CY01Y and CY03Y comparisons. The horizontal coordinates in the plot indicate the fold change of gene expression in the two groups of treatment (log2FoldChange), and the vertical coordinates indicate the significance level of the difference in gene expression in the two groups of treatment (−log10pvalue). Up-regulated genes are indicated by red dots, and down-regulated genes are indicated by green dots. **(D)** The top 30 GO terms of 192 common differential genes. **(E)** KEGG enrichment of the 166 genes decreased in expression year by year.

To examine gene expression disparities, read counts from the 18 samples underwent DESeq2 analysis. Our analysis identified 4,035 differentially expressed genes between 1-year-old and 2-year-old plants, 5,891 differentially expressed genes between 1-year-old and 3-year-old plants, and 920 differentially expressed genes between 2-year-old and 3-year-old plants at *p* < 0.05 (Figure 2). Among them gene, 2,132 (52.8%) were up-regulated and 1,903 (47.2%) were down-regulated in annual and biennial *D. nobile* (Figure 2A). Similarly, the expression of 3,624 (61.5%) genes was up-regulated and 2,267 (38.5%) genes were down-regulated in one-year plants as compared to three-year plants (Figure 2B). However, fewer differential genes were identified between 2-year and 3-year plants, with only 748 (81.3%) genes up-regulated and 172 (18.7%) genes down-regulated in expression (Figure 2C). A Venn diagram of the commonly expressed differential genes between the different years of *D. nobile* stems revealed 192 common differential genes in the three sets of samples (Figure 1D).

For a deeper understanding of the functional distinctions among differentially expressed genes, we conducted Gene Ontology (GO) term enrichment analysis. Twenty-five genes were annotated to 154 biological processes, encompassing external encapsulating structure organization, cell wall organization, glucan metabolic processes, carboxylic acid metabolic processes, and others. Three genes were annotated to 16 cellular components including cell wall, external encapsulating structure, apoplast extracellular region, etc. Notably, differential genes were predominantly localized in the cell wall and organelles within the cytoplasm. Furthermore, 44 genes were annotated to 93 molecular functions, such as GTPase activity, heme binding, tetrapyrrole binding, transferase activity, enzyme inhibitor activity, glucosyltransferase activity, and others (Supplementary Table S2). Among the top 30 annotated GO terms, molecular function accounted for 53 percent while biological processes accounted for 47 percent (Figure 2D). Analyzing the 192 screened genes (Supplementary Table S3) through heatmap clustering, we observed that 166 genes exhibited a gradual decrease in expression year by year, while only 10 genes displayed a gradual increase in expression with age. The highest expression was observed in the 2-year-old plants, consistent with another 16 genes (Supplementary Figure S1). Subsequently, KEGG enrichment analysis of the three groups of differential genes revealed that group A differential genes were predominantly enriched in 32 biological pathways, including Phagosome, Phenylpropanoid biosynthesis, Phenylalanine metabolism, Fatty acid degradation, alpha-Linolenic acid metabolism, Plant hormone signal transduction, Sesquiterpenoid and triterpenoid biosynthesis, Biosynthesis of unsaturated fatty acids, and others (Figure 2E).

### 3.2 Endophytic fungal community composition in the stems shifts along the age axis

**Figure 3:**
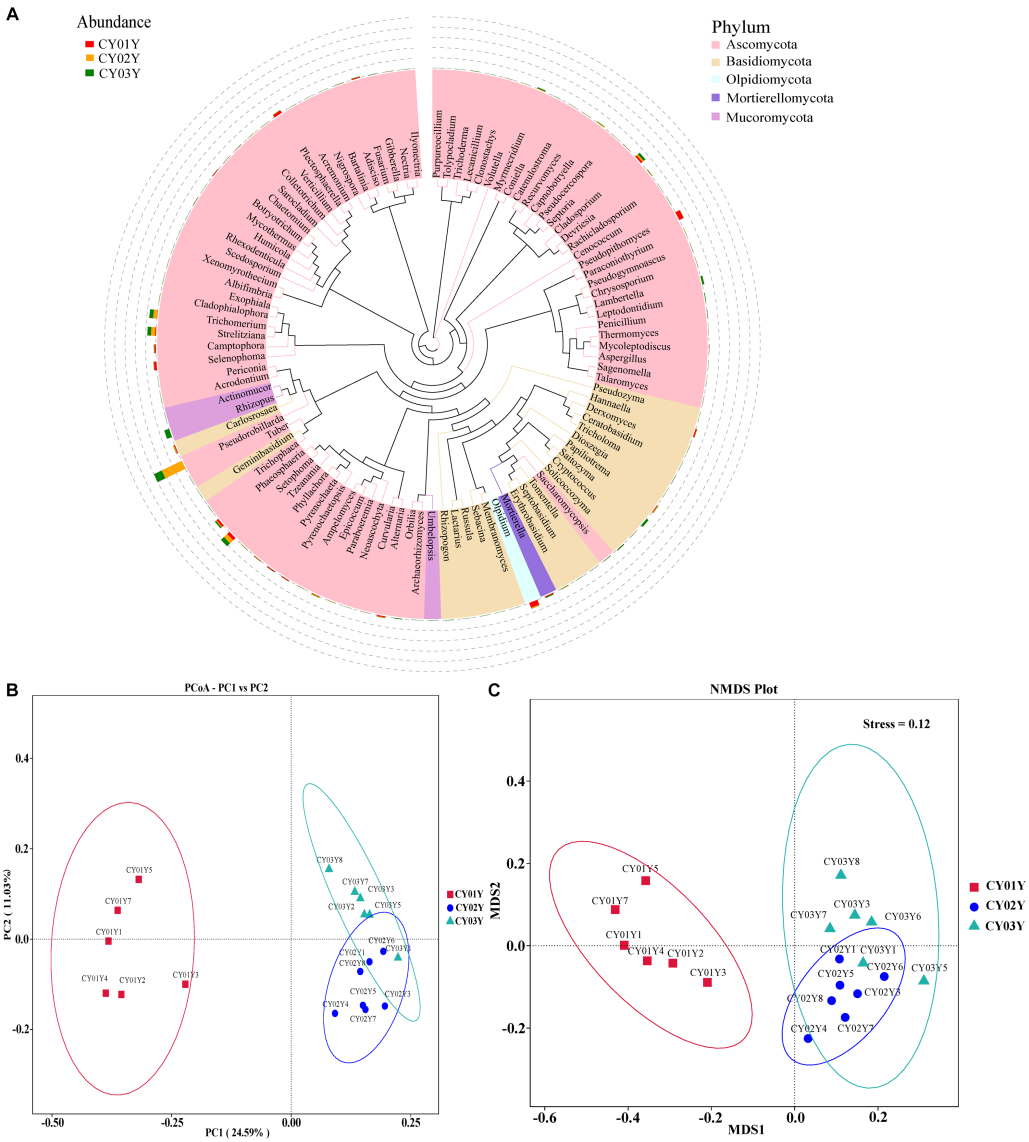
Endophytic fungal community composition outline in the stems of *D. nobile*. **(A)** Top 100 genera in species evolutionary trees at genus level. The colors of the branches and sectors indicate different phyla, and the stacked bar graphs on the exterior of the sector ring indicate information on the distribution of abundance of the genera in different samples. **(B)** Principal Co-ordinates Analysis (PCoA) of the 1,817 most abundant ASVs with unweighted UniFrac. **(C)** Nonmetric Multidimensional Scaling (NMDS) of the 1,817 most abundant ASVs with unweighted UniFrac. The samples were clustered based on different ages (CY01Y, CY02Y or CY03Y).

**Figure 4:**
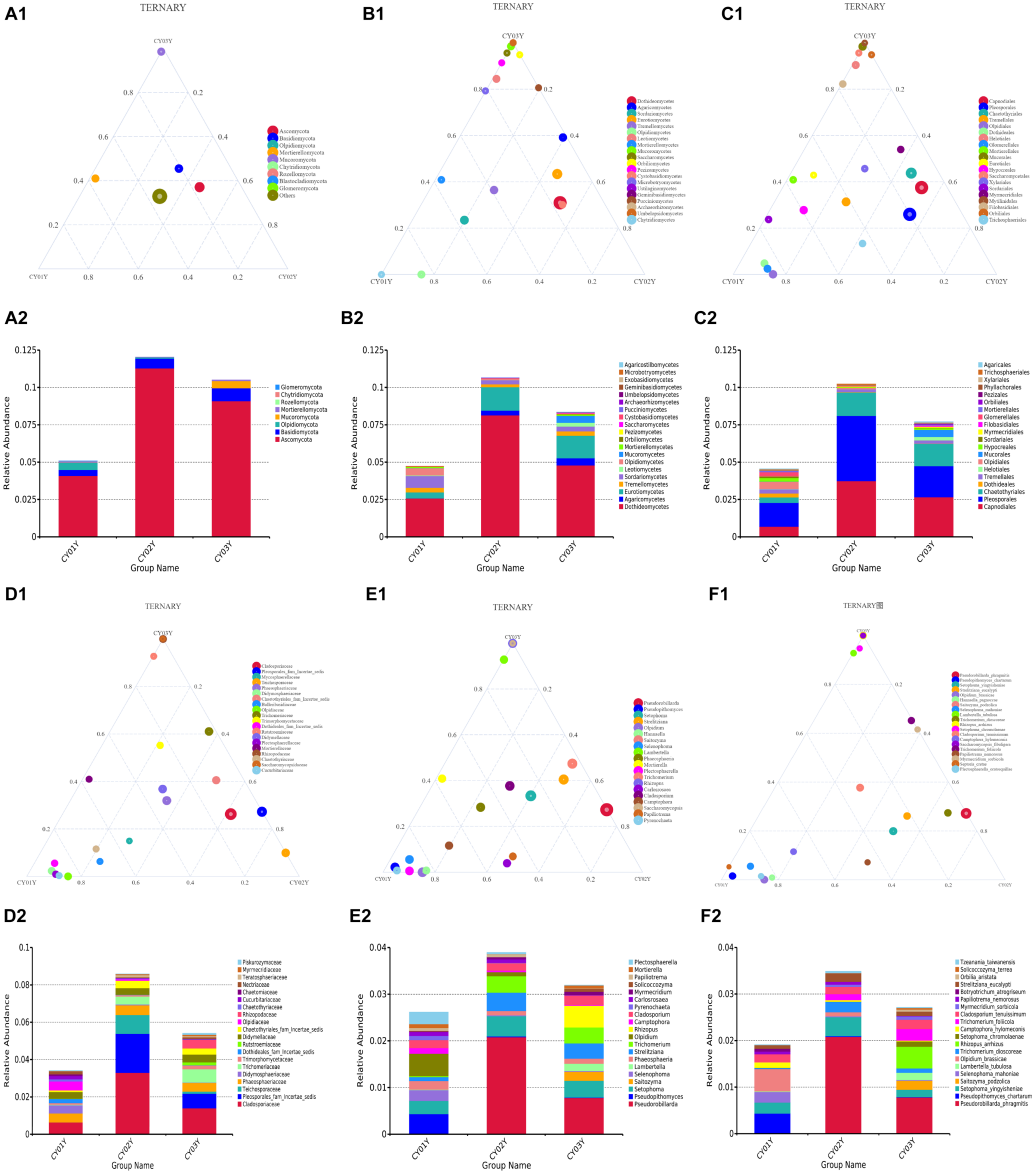
The ternaryplots and relative abundance accumulation diagram of endophytic fungi in *D. nobile* at different levels of categorization. **(A1)** The ternaryplot at the phylum level. **(A2)** The relative abundance accumulation diagram at the phylum level. **(B1)** The ternaryplot at the class level with top 20 classes. **(B2)** The relative abundance accumulation diagram at the class level. **(C1)** The ternaryplot at the order level with top 20 orders. **(C2)** The relative abundance accumulation diagram at the order level. **(D1)** The ternaryplot at the family level with top 20 families. **(D2)** The relative abundance accumulation diagram at the family level. **(E1)** The ternaryplot at the genus level with top 20 genus. **(E2)** The relative abundance accumulation diagram at the genus level. **(F1)** The ternaryplot at the species level with top 20 species. **(F2)** The relative abundance accumulation diagram at the species level.

In this study, we thoroughly examined the distribution of endophytic fungal taxa in *D. nobile* stems across different age groups through internal transcribed spacer sequencing, complemented by a detailed analysis of the fungal microbiomes (Methods). The systematic sampling approach (Figure 1A) was validated by rarefaction analysis, affirming the sequencing depth was sufficient to capture the majority of endophytic fungal members in all 18 stem samples (Supplementary Figure S2). A total of 1.72 million Effective Tags underwent analysis using QIIME2, leading to the identification of 1,817 Amplicon Sequence Variants (ASVs) at a 100% identity level. The overall analysis demonstrated discernible distinctions among the three age groups (Figures 3A, 4A2).

**Figure 5:**
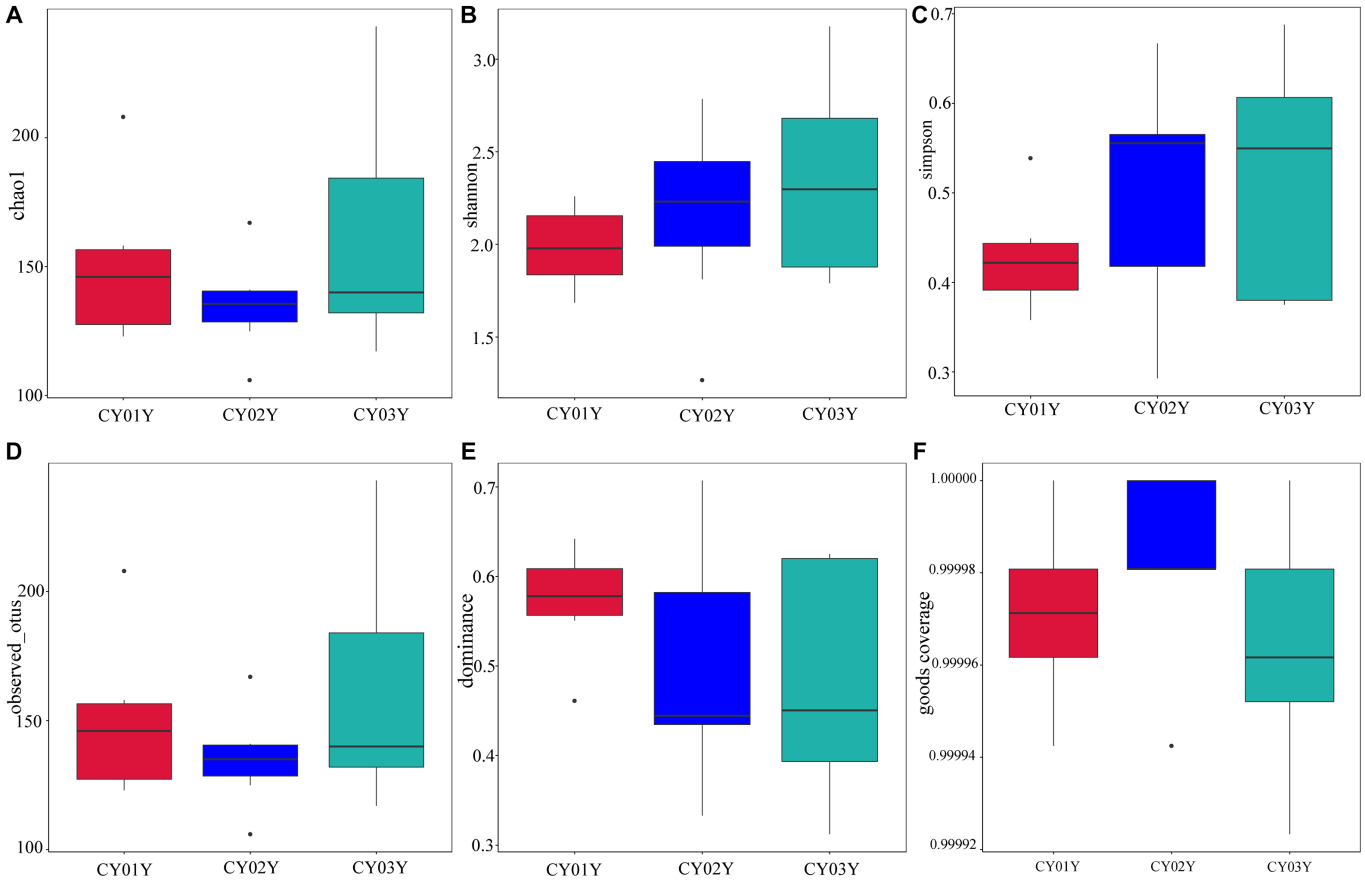
Species alpha diversity of stem-endophytic fungi in *D. nobile*. **(A)** The Chao 1 index of the endophytic fungi and Tukey’s test showed no significant difference (*p* > 0.05), indicating that the number of species in stems is similar at different ages. **(B)** The Shannon index of endophytic fungi. **(C)** The Simpson diversity index showed no significant difference between them (*p* > 0.05). **(D)** The observed OTUs of endophytic fungi in *Dendrobium nobile.* **(E)** Dominance of different samples, the indices are all less than 1, indicating that the community has good species uniformity. **(F)** Good coverage index shows the higher sequencing coverage.

Principal Co-ordinates Analysis (PCoA) (Figure 3B) and Nonmetric Multidimensional Scaling (NMDS) (Figure 3C) of the 1,817 most abundant ASVs based on unweighted UniFrac and nonphylogenetic Bray-Curtis metrics (Adonis *p* value =0.001) unveiled a notable shift in fungal community composition within *D. nobile* stems across the age gradient. Both PCoA and NMDS plots highlighted distinct clustering based on age groups (CY01Y, CY02Y, or CY03Y). Alpha diversity metrics, including Chao1, observed OTUs, Shannon, and Simpson indices, indicated a balanced overall biodiversity of endophytic fungi across all samples (Figure 5).

We further investigated taxonomic differences in abundance among the endophytic microbiomes of CY01Y, CY02Y, and CY03Y plants using phyloseq. Eight phyla and 20 classes, orders, and genera were selected for analysis at different taxonomic ranks. Among them, 20 highly abundant ASVs exhibited significant differences between CY01Y, CY02Y, and CY03Y (Figure 4). Ternary plots and abundance accumulation diagrams of endophytic fungi in *D. nobile* stems across different ages were generated to visualize dominant taxa variations (Figure 4). Ascomycota exhibited the highest abundance in all samples, with significant enrichment in 2-year-old and 3-year-old *D. nobile* stems. Basidiomycota displayed a similar trend. Mucoromycota were particularly abundant in 3-year-old samples, whereas Mortierellomycota were nearly absent in 2-year-old samples (Figures 4A1,A2).

**Figure 6:**
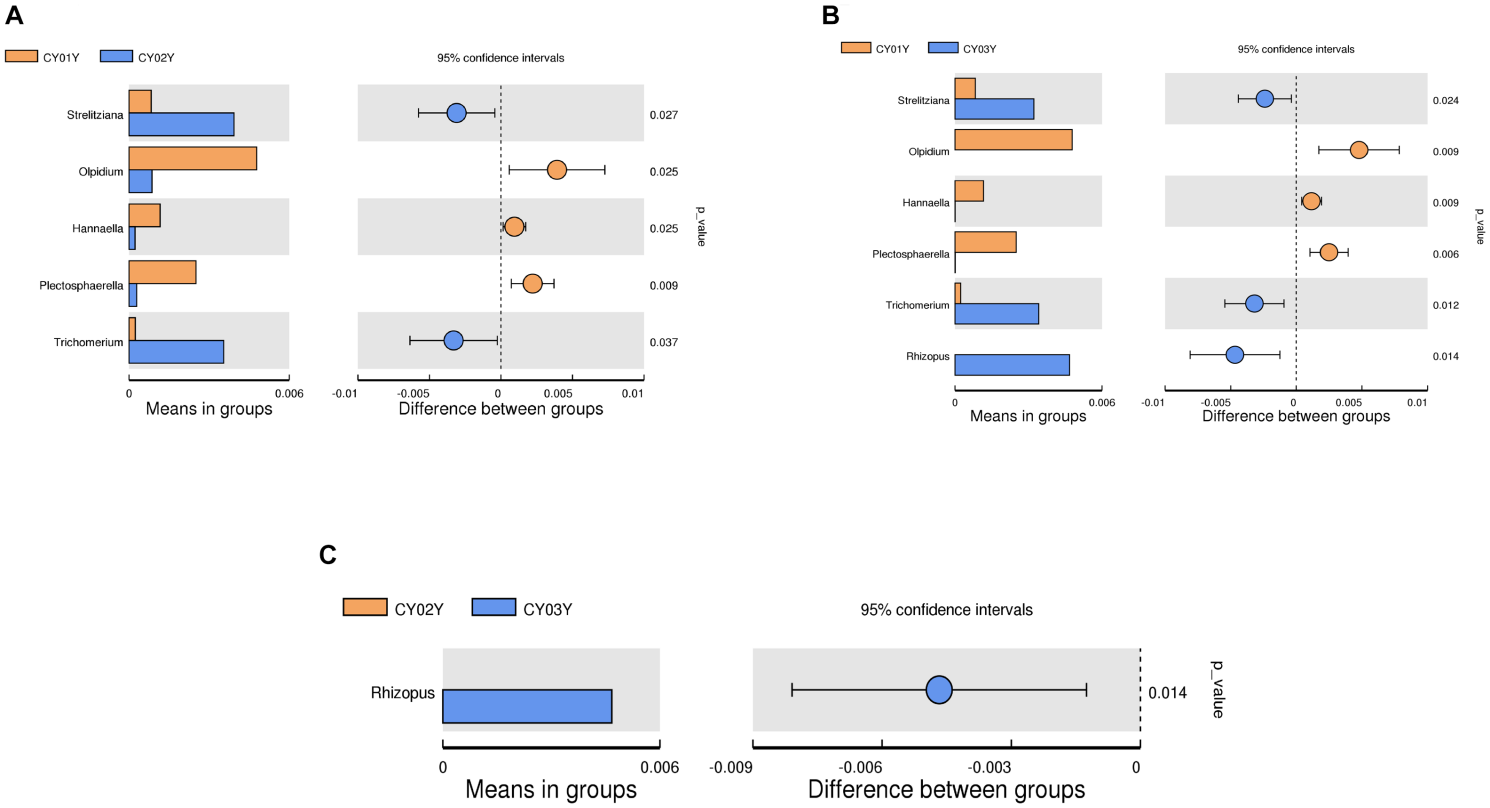
T-test of endophytic fungi in *D. nobile* at different ages at genus level. Each bar in the figure represents the mean value in each group for genera with significant differences in abundance between groups. The right panel shows the confidence level of intergroup differences. The leftmost endpoint of each circle in the figure represents the lower limit of the 95% confidence interval of the difference in means, and the rightmost endpoint of the circle represents the upper limit of the 95% confidence interval of the difference in means. The center of the circle represents the difference in means, and the circle color represents the value of p of the significance test for between-group differences for the corresponding differential taxa. **(A)** T-test of CY01Y and CY02Y. **(B)** T-test of CY01Y and CY03Y. **(C)** T-test of CY02Y and CY03Y.

At the genus level, *Pseudorobillarda* showed significant enrichment in 2-year-old and 3-year-old *D. nobile* stems, with markedly higher abundance in the 2-year-old plants compared to the 3-year-old ones. Conversely, *Pseudopithomyces* was enriched exclusively in 1-year-old samples and absent in 2- and 3-year-old samples. Several fungi were specifically enriched in 1-year-old samples, including *Pyrenochaeta*, *Plectosphaerella*, *Selenophoma*, *Olpidium*, and *Hannaella*, while no fungi were specifically enriched in 2-year-old stems. Three genera, *Rhizopus*, *Lambertella*, and *Saccharomycopsis*, exhibited higher abundance in 3-year-old samples compared to the other groups (Figures 4E1,E2). The T-test results demonstrated higher similarity in endophytic fungi between 2-year-old and 3-year-old plants, while 1-year-old plants exhibited greater dissimilarity, aligning with the differences observed between 2-year-old and 3-year-old plants. Fungal genera significantly enriched in annuals were *Olipidium*, *Hannaella*, and *Plectospherella*, whereas endophytic fungi *Strelitziana* and *Trichomerium* were most enriched in biennials. Perennials were enriched in endophytes, including *Rhizopus*, with only a single addition compared to the biennials, while the rest remained consistent (Figure 6).

**Figure 7:**
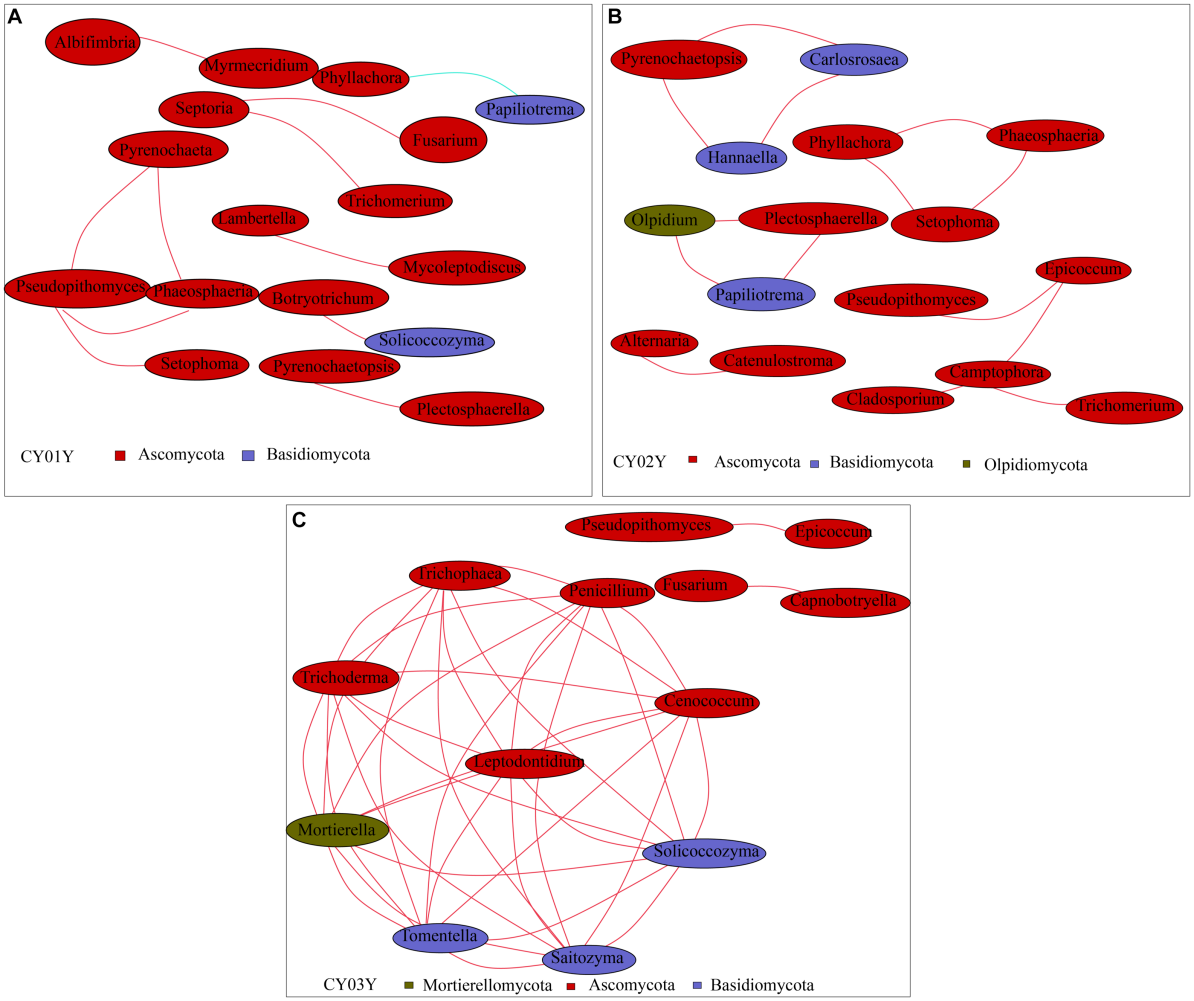
Co-occurrence network of age-associated ASVs selected. Different nodes represent different genera, node size represents the average relative abundance of the genus, nodes of the same phylum are of the same color (as shown in the legend), the thickness of the lines between the nodes is positively correlated with the absolute value of the correlation coefficient of the species interactions, and the color of the lines corresponds to the positive and negative correlation (red positive correlation, blue negative correlation). **(A)** Co-occurrence network of CY01Y. **(B)** Co-occurrence network of CY02Y. **(C)** Co-occurrence network of CY03Y. The key parameters in Network Diagram such as Network Diameter (ND), Modularity Degree (MD), Clustering Coefficient (CC), Graph Density (GD), Average Degree (AD). Average Path Length (APL) are shown in Supplementary Table 4.

To probe into the relationships between stem genotype and age-dependent fungal ASVs in endophytic fungi, we constructed a co-abundance network. In line with PCA data, endophytic fungal ASVs exhibited intricate networks, providing insights into interactions between fungal communities at different age states. Notably, distinctive differences were observed in the community structures of microbes associated with 1-year-old stems versus those associated with 2- or 3-year-old stems. For microbes linked with 1-year-old stems, Spearman correlation analysis identified 17 species with a correlation above a threshold of 0.2 (*p* < 0.01; Figure 7A). Positive correlations were observed, such as *Pseudopithomyces* with *Pyrenochaeta*, *Phaeosphaeria*, and *Setophoma*, as well as *Septoria* with *Trichomerium* and *Fusarium*. Additionally, there were pairwise correlations between *Albifimbria* and *Myrmecridium*, *Lambertella* and *Mycoleptodiscus*, and *Botryotrichum* and *Solicoccozyma*. However, *Phyllachora* exhibited a negative correlation with *Papiliotrema*. For microbes associated with 2-year-old stems, Spearman correlation analysis revealed 16 taxa with a correlation above a threshold of 0.2 (*p* < 0.01; Figure 7B). Positive correlations were observed among *Pyrenochaetopsis*, *Carlosrosaea*, and *Hannaella*; *Phyllachora*, *Phaeosphaeria*, and *Setophoma*; and *Olpidium*, *Plectosphaerella*, and *Papiliotrema*. Additionally, there was a positive correlation between *Alternaria* and *Catenulostroma*, as well as *Epicoccum* with *Pseudorobillarda*, *Camptophora*, and *Cladosporium*. Furthermore, *Camptophora* showed a positive correlation with *Trichomerium*, while *Alternaria* showed a positive correlation with *Catenulostroma*. Thirteen species exhibited significant Spearman correlation with 3-year-old stems (Spearman >0.2, *p* < 0.05; Figure 7C). Among them, nine taxa (*Tomentella, Trichoderma*, *Mortierella*, *Tomentella*, *Saitozyma*, *Solicoccozyma*, *Cenococcum*, *Penicillium*) positively correlated with each other. The other two taxa groups *Pseudopithomyces* and *Epicoccum*, *Fusarium* and *Capnobotryella* also positively correlated.

### 3.3 Association of the stem transcriptome and endophytic fungi across different ages

**Figure 8:**
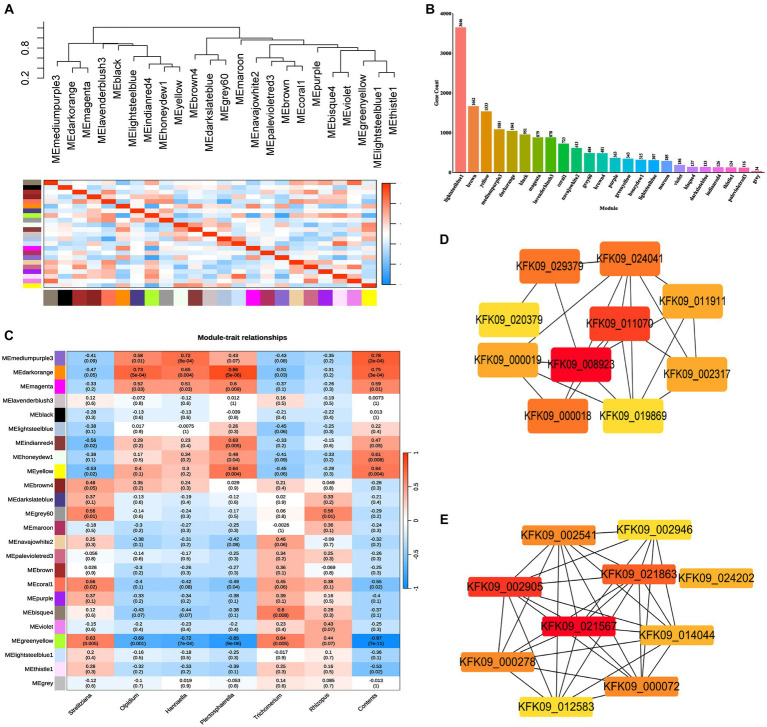
WGCNA analysis of all genes in the unigenes of the RNA-Seq data (weighting factor β = 10 and Height of Clustering of module eigengenes was 0.3). **(A)** Clustering dendrogram of all 18 samples. **(B)** Histogram of the number of genes in each module. **(C)** Pearson correlation coefficients between the 24 modules and dominant endophytic fungi and the contents of dendrobine. **(D)** The hub genes interaction network in cytoscape filtered by cytoHubba of mediumpurple3 module. **(E)** The hub genes interaction network in cytoscape filtered by cytoHubba of darkorange module.

The stem RNA-sequencing (RNA-seq) reads and microbial amplicon reads underwent stringent filtering to establish significant correlations of the factors ages, genes, and fungi between the datasets, thereby associating gene expression with fungi ASV abundance. Utilizing WGCNA analysis on all genes in the unigenes of the RNA-Seq data, 24 modules were delineated (weighting factor β = 10 and Height of Clustering of module eigengenes was 0.3). Among these, 16,441 genes were enriched in 23 modules, while 34 genes were not enriched in these modules and were cataloged as gray modules, resulting in a total of 24 modules (Supplementary Table S5; Figure 8A). The distribution of genes within each module is illustrated in Figure 8B.

Pearson correlation coefficients and environmental factors were computed for the Module Eigengenes (MEs) of all modules to identify modules associated with environmental factors (Figure 8C). The mediumpurple3 module exhibited a significant correlation with the endophytic fungal genera *Olpidium* (*R* = 0.58, *p* = 0.01), *Hannaella* (*R* = 0.72, *p* = 8e-04), and *Plectosphaerella* (*R* = 0.43, *p* = 0.07). Similarly, the darkorange module demonstrated a significant correlation with the endophytic fungal genera *Olpidium* (*R* = 0.73, *p* = 5e-04), *Hannaella* (*R* = 0.65, *p* = 0.004), and *Plectosphaerella* (*R* = 0.86, *p* = 5e-06). Additionally, the greenyellow module exhibited a significant correlation with the endophytic fungal genera *Strelitziana* (*R* = 0.63, *p* = 0.005) and *Trichomerium* (*R* = 0.64, *p* = 0.005), while showing a significantly lower correlation with the endophytic fungal genus *Rhizopus* (Figure 8C).

**Figure 9:**
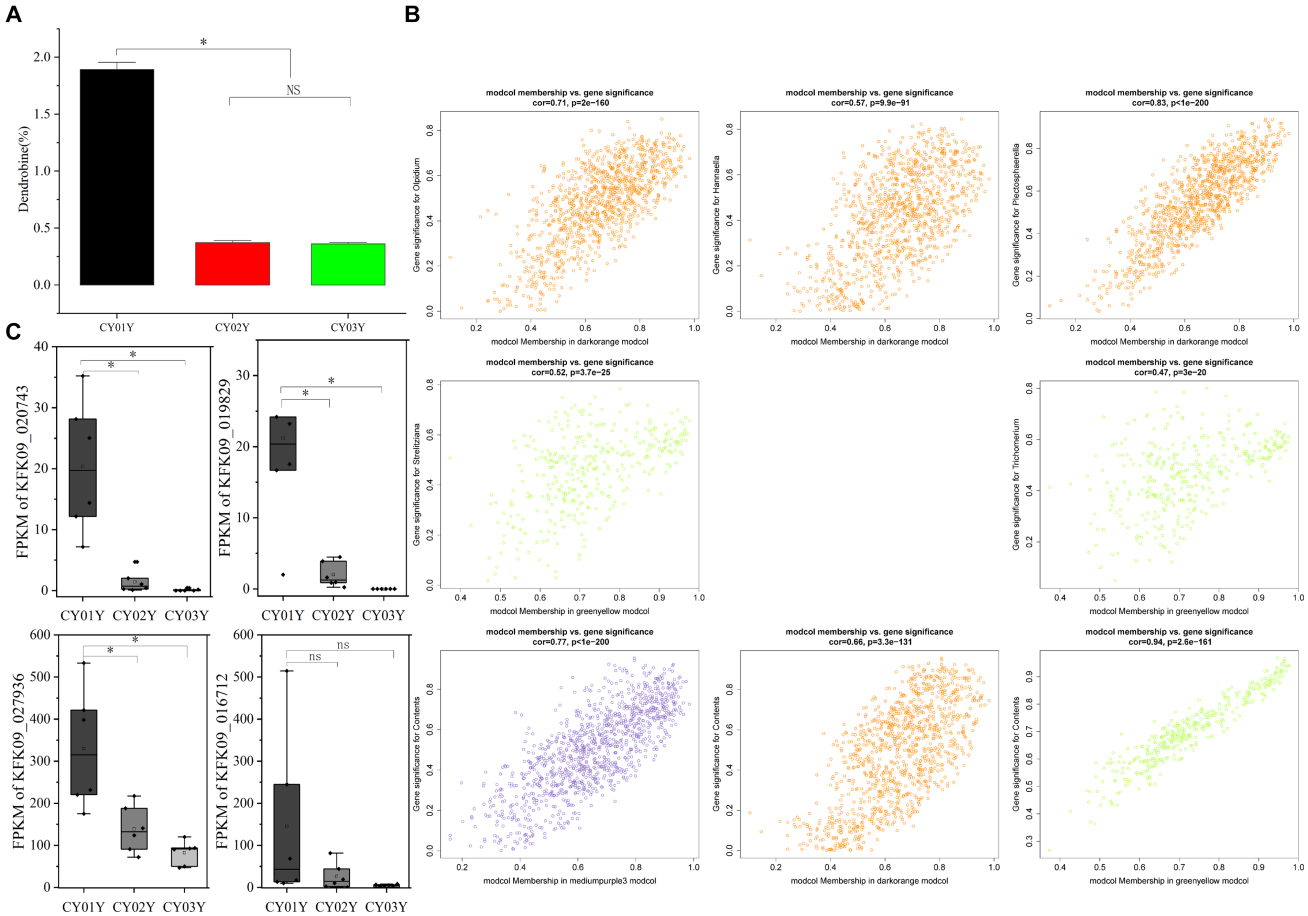
Dendrobine content and relationship with endophytic fungi and the plant transcriptome. **(A)** Contents of dendrobine along the timeline. **(B)** Module Membership vs. Gene Significance of darkorange, greenyellow and mediumpurple3 modules. **(C)** The expression of four differential genes enriched in phytohormone signaling (*p < 0.05).

Subsequently, module membership (MM) was assessed to determine the correlation between genes and specific modules. Correlation analyzes were conducted between the Gene Significance (GS) of significantly different endophytic fungi and the MM of each modular gene to ascertain if the MM valus closely aligned with these endophytes, as well as dendrobine content. The outcomes revealed that in the darkorange module, the correlation coefficient between GS for *Olpidium*, *Hannaella*, and *Plectosphaerella* and MM was the highest. The correlation coefficients were cor = 0.71, *p* = 2e-160; cor = 0.57, *p* = 9.9e-91; cor = 0.83, *p* < 1e-200. Furthermore, in the greenyellow module, the correlation coefficients between the GS for endophytic fungi *Strelitziana* and *Trichomerium* and MM were the highest (cor = 0.52, *p* = 3.7e-25; cor = 0.47, *p* = 3e-20; Figure 9B).

**Figure 10:**
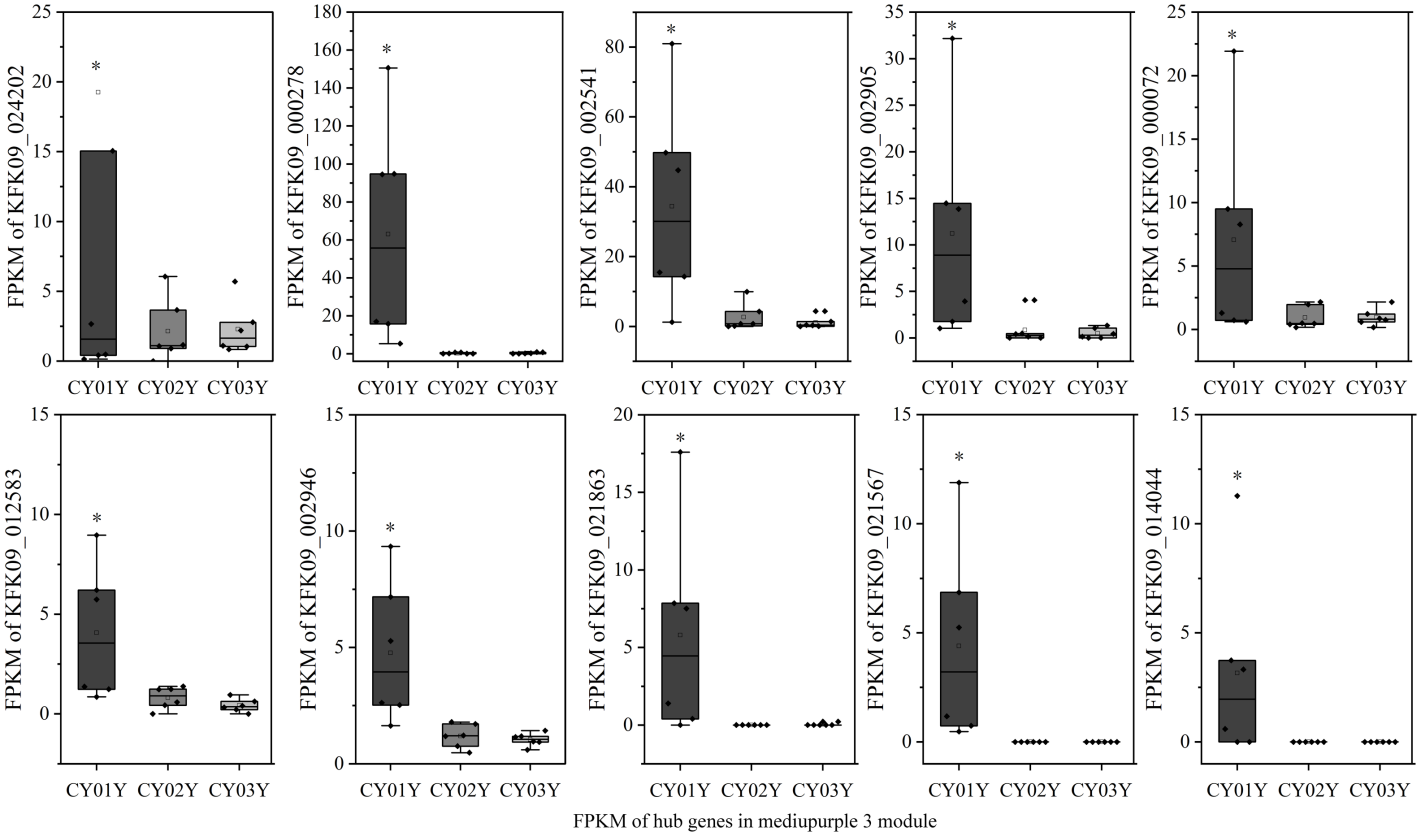
The expression graph of 10 genes in the mediumpurple3 module. The diagram showed the gene expression is significantly higher in annuals than in 2- and 3-year olds (*p < 0.05).

To elucidate hub genes associated with the predominant endophytic fungi in 1-year-old *D. nobile* stems, the mediumpurple3 and darkorange modules were filtered based on Gene Significance (GS) and Module Membership (MM) values with a threshold of 3. The selected genes were then imported into Cytoscape, and the top 10 hub genes were identified using the cytoHubba plugin (Figures 8D,E). Among these genes, KFK09_024202 is a cell wall structural protein gene, KFK09_000278 is a CRIB domain-containing protein gene, KFK09_002541 is an mRNA of laccase-3, KFK09_000072 is an mRNA of probable polygalacturonase, KFK09_012583 is a HIP41 ARATH Heavy metal-associated isoprenylated plant protein gene, KFK09_002946 is a TOR1 ARATH Microtubule-associated protein gene, KFK09_021863 is a PMI11_ARATH Pectinesterase inhibitor coding mRNA, and KFK09_021567 is a KATAM_ORYSJ Xyloglucan galactosyltransferase gene (Figure 10).

**Figure 11:**
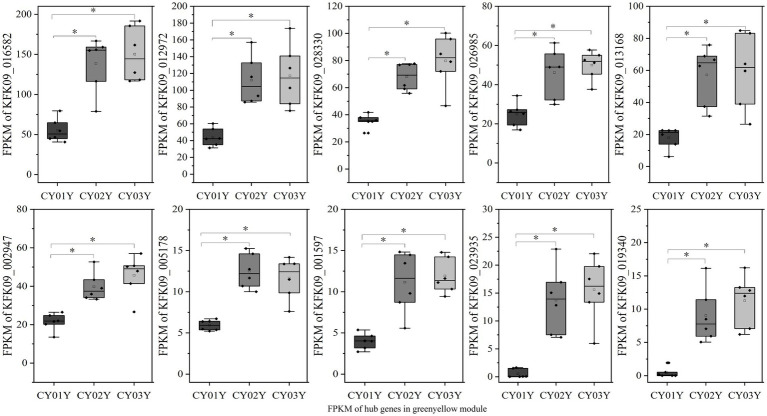
The expression graph of 10 genes in the greenyellow module. The graph shows that the expression of genes increases with the age of the plant (*p < 0.05).

For an in-depth analysis of the association of the greenyellow module with dominant endophytic fungi in 2- and 3-year-old *D. nobile*, 10 hub genes in the greenyellow module were selected based on GS and MM values, with a focus on MM > 0.8 and GS > 0.2. These hub genes exhibited significantly higher expression in 2- and 3-year-old plants compared to 1-year-old plants. Additionally, they exhibited a consistent trend of expression with the endophytic fungi *Trichomerium* and *Strelitziana* in *D. nobile* stems (Figure 11).

Consequently, it can be inferred that the gene expression modules significantly associated with the dominant endophytes of *D. nobile* are the mediumpurple3 and darkorange modules, correlating with different plant ages. The predominant fungi in young 1-year-old stems belong to the genera *Olpidium*, *Hannaella*, and *Plectosphaerella*, exhibiting positive correlations with the mediumpurple3 and darkorange modules. Conversely, the dominant endophytic fungi expressed in older 2- and 3-year-old stems pertain to *Trichomerium* and *Strelitziana* species, and are significantly and positively correlated with the greenyellow module.

### 3.4 Age-dependent changes in the stem transcriptome and endophytic fungi are synchronized with properties of synthetic dendrobine in plants

Furthermore, we investigated whether the decline in dendrobine content with age is associated with age-specific transcriptome characterization in interaction with endophytic fungi. The 1-year-old stems of *D. nobile* exhibited the highest accumulation of dendrobine (Figure 9A). Through WGCNA analysis of the transcriptomes of Dendrobium stems of three different ages, we identified four modules - mediumpurple3 (*R* = 0.78, *p* = 2e-04), darkorange (*R* = 0.75, *p* = 3e-04), honeydew1 (*R* = 0.61, *p* = 0.008), and yellow (*R* = 0.64, *p* = 0.004) - that demonstrated a significant positive correlation with dendrobine content. In contrast, the greenyellow module (*R* = −0.97, *p* = 7e-11) displayed a significant negative correlation with dendrobine content. Subsequently, we conducted a correlation analysis between the GS of dendrobine content and MM of genes in each module to examine their relationship with dendrobine content. The highest correlation coefficients between GS of dendrobine content and MM were observed in the greenyellow, followed by the mediumpurple3 and darkorange modules, with correlation coefficients of cor = 0.94, *p* = 2.6e-161; cor = 0.77, *p* = 1e-200; and cor = 0.66, *p* = 3.3e-131 (Figure 9B). Given its significant negative correlation with dendrobine, the greenyellow module was not further analyzed.

The mediumpurple3 and darkorange modules were then subjected to GO functional enrichment. The mediumpurple3 module was primarily enriched for processes related to carbohydrate metabolism and transferase activity, while the darkorange module exhibited enrichment in processes associated with transferase activity and oxidoreductase activity (Supplementary Figure S3). However, only a few genes in the modules related to endophytic fungi showed significant enrichment in GO functions. This indicates that dissimilarity in gene expression does not always correspond to functional diversity.

Specifically, among the hub genes identified in these modules, only KFK09_002541, KFK09_000072, and KFK09_021863 were enriched in GO functional categories related to oxidation–reduction processes, carbohydrate metabolism, and enzyme inhibitor activity. Notably, no significant direct correlation with the dendrobine synthesis pathway was observed. Since alkaloid synthesis has been reported to correlate with the phytohormone pathway (Yan et al., 2019), we also examined four differential genes enriched in phytohormone signaling at different age gradients (gene-KFK09_020743, gene-KFK09_019829, gene-KFK09_027936, gene-KFK09_016712) categorized in the mediumpurple3 module. While these genes were not selected as core genes by WGCNA, they were listed as hub genes due to their functional significance (Figure 9C).

**Figure 12:**
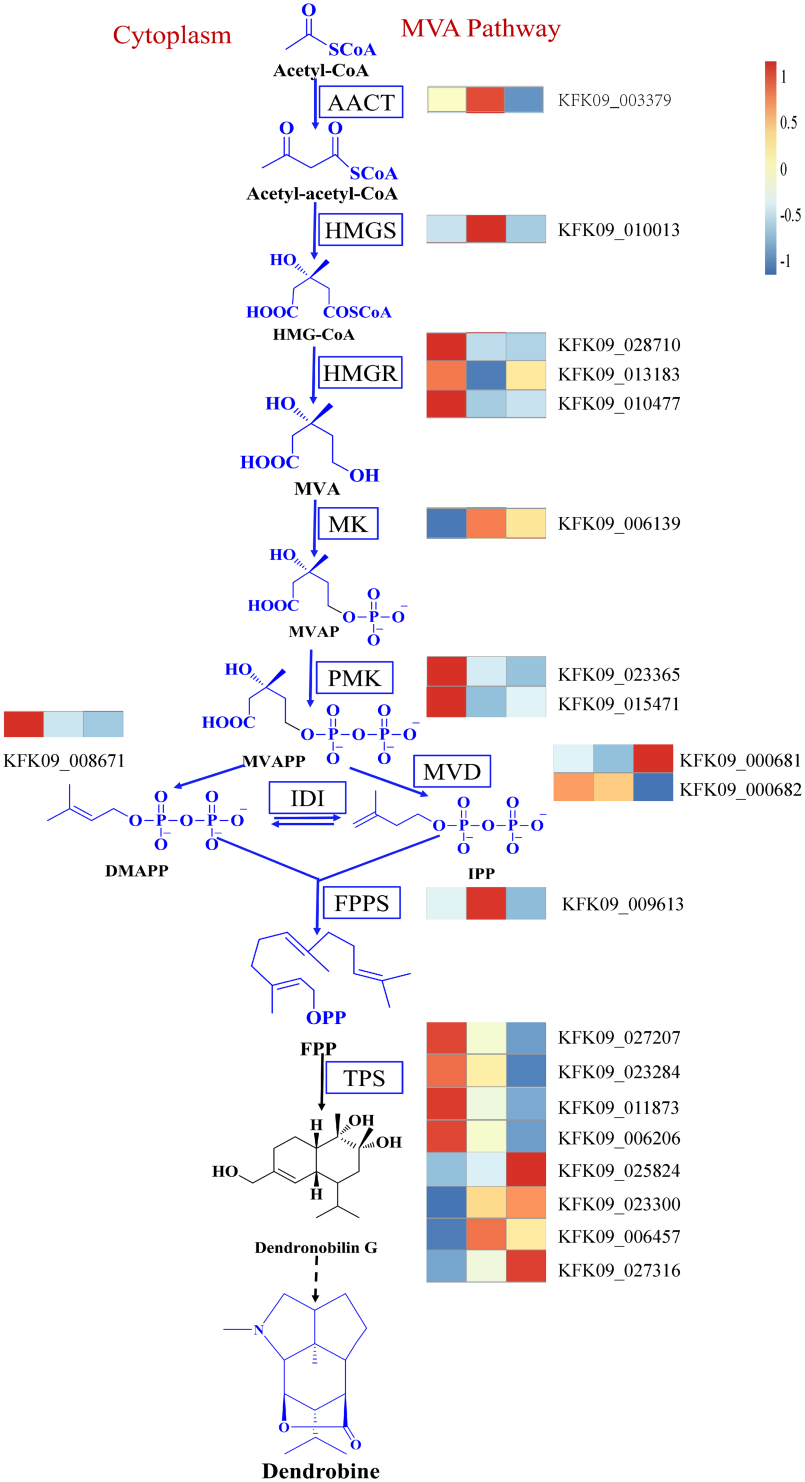
The MVA pathway of dendrobine and changes in key genes of the pathway. AACT, Acetoacetyl-CoA thiolase; HMGS, 3-hydroxy-3-methylglutaryl-coenzymeA synthase; HMGR, HMG-CoA reductase; MK, Mevalonate kinase. PMK, Phosphomevalonate kinase; MVD, Mevalonate diphosphate decarboxylase; IDI, Isopentenyl diphosphate isomerase; FPPS, Farnesyl diphosphate synthase; TPS, Terpenoid synthase.

In addition, it’s crucial to consider the reported changes in the expression of genes involved in the dendrobine synthesis pathway. The MVA pathway and the MEP pathway are the most documented biogenic pathways for dendrobine synthesis (Gong et al., 2021). Our data mining identified relevant genes in the MVA pathway, including acetoacetyl-CoA thiolase (AACT), 3-hydroxy-3-methylglutaryl-CoA synthase (HMGS), mevalonate kinase (MK), phosphomevalonate kinase (PMK), mevalonate diphosphate decarboxylase (MVD), farnesyl diphosphate synthase (FPPS), and others. Genes that displayed a similar trend to dendrobine content included the HMGR genes KFK09_028710, KFK09_013183, KFK09_010477, as well as the PMK genes KFK09_023365, KFK09_015471, the IDI gene KFK09_008671, and some of the TPS genes KFK09_027207, KFK09_023284, KFK09_011873, and KFK09_006206 (Figure 12). Although none of the relevant genes in the MVA pathway were enriched in the mediumpurple3 and darkorange modules, their significance should not be overlooked. This suggests that the variation in dendrobine content across different years cannot be attributed solely to any one pathway. Furthermore, the subsequent biological processes involved in the formation of dendrobine from the terpene skeleton remain unclear (Chen et al., 2021), indicating that genetic changes in the MVA pathway and MEP pathway do not constitute the sole correlation with dendrobine synthesis.

## 4 Discussion

Since ancestral plants settled on land 450 million years ago, they have been interacting with microorganisms to form a “holobiont” (Hassani et al., 2018). These ubiquitous interactions between microbial communities and their host plants often shape host phenotypes and regulate metabolite synthesis (Sharma et al., 2021). Plants have evolved the ability to produce a large number of unique metabolites, and this metabolic diversification is likely driven by the need to adapt to different environments (Chae et al., 2014). One of the key environmental factors affecting plant health and adaptability is the host microbiota (Bulgarelli et al., 2012). Recent studies reveal an additional role for the plant-specialized metabolite camalexin in facilitating host-inter-root microbial interactions (Huang et al., 2019). However, how the microbial-host maintains homeostasis after perturbation remains poorly understood (Ma et al., 2021). Thus, the interaction between endophytic fungi and host has attracted wide attention, because they are a group of symbiotic fungi that colonize plant tissues and organs without causing apparent disease (Clay, 2004), but have the ability to reshape plant characteristics (Yan et al., 2019). There are two sides to every story. Endophytic fungi can provide various benefits to the host plant, such as promoting growth (Koberl et al., 2013; Shan et al., 2021), enhancing stress tolerance, inducing resistance (Ma et al., 2021) and producing bioactive compounds (Ancheeva et al., 2020). However, they also have negative effects on the host plant, conversion of endophytes from symbiotic to parasitic state (Thrall et al., 2007). Therefore, the interaction between endophytic fungi and host plant is complex and dynamic, and needs to be explored and understood in depth at different levels and scales.

*Dendrobium nobile* is a traditional Chinese medicinal herb with various pharmacological effects, mainly derived from its main active ingredient, dendrobine. However, the content of dendrobine in *D. nobile* varies greatly with the age of the plant, and the underlying mechanism is still unclear (Gong et al., 2021). In this study, we observed significant shifts in the composition and abundance of endophytic fungi in the stems of *D. nobile* along the age axis, which correlated strongly with changes in the stem transcriptome. These findings are consistent with those of other studies on plants (Liu et al., 2017). Based on these observations, we hypothesized that age-dependent changes in the stem transcriptomes might be responsible for the observed variations in endophytic fungi. Several fungal genera, such as *Olpidium*, *Hannaella*, *Plectosphaerella*, *Strelitziana* and *Trichomerium*, were associated with different transcriptomic modules and dendrobine content. Meanwhile, some genes in plant hormone signaling and alkaloid biosynthesis pathways were differentially expressed among different ages and correlated with endophytic fungi and dendrobine content. Our results suggest that endophytic fungi may play an important role in regulating the stem transcriptome and dendrobine synthesis in *D. nobile* along the age gradient.

The co-occurrence network analysis revealed that the endophytic fungal community in the stems of *D. nobile* had a modularly structured according to different age groups. For instance, the 1-year-old *D. nobile* had a unique fungal community and corresponding transcriptomic module, which showed the highest correlation with *Olpidium*, *Hannaella*, and *Plectosphaerella* at the genus level compared to all other samples. *Olpidium* is known to act as a vector for plant viruses, infecting many plants with a wide range of hosts (Lay et al., 2018). At the level of species, there are *Olpidium brassicae* and *Olpidium virulentus* in the stems. *O. brassicae* and other *Olpidium* species sometimes form a large part of the fungal community in other plant roots or rhizospheres (Tkacz et al., 2015). Hannaella, which is a basidiomycetous yeast genus belonging to the order Tremellales, phylum Basidiomycota, had the highest abundance in *Alpinia zerumbet* (Yan et al., 2022) and *Salvia miltiorrhiza* (Chen et al., 2018). There are *Hannaella pagnoccae*, *Hannaella sinensis*, *Hannaella oryzae*, *Hannaella surugaensis*, *Hannaella luteola* and other species in our samples, and they all have been described in the *Hannaella* genus. Widespread on the leaf surfaces of various plants, including rice, wheat, and fruit trees (Han et al., 2017). *H. oryzae* was thought to play an important role in plant growth promotion and biocontrol as an important phyllosphere inhabiting yeast (Li Q. et al., 2021). The genus *Plectosphaerella* is the largest genus in the family Plectosphaerellaceae. Some species are plant pathogens, whereas others are soil-borne (Zhang et al., 2019). In the stem of *D. nobile*, several species were detected including *Plectosphaerella oratosquillae*, *Plectosphaerella oratosquillae* and *Plectosphaerella alismatis*. Most species of *Plectosphaerella* are pathogens causing large losses in agriculture reportedly (Yang et al., 2021). Therefore, according to the analysis, *Hannaella* is one of the endophytes instructive by subsequent studies. Moreover, module greenyellow had a significantly high correlation with fungus *Strelitziana*, *Trichomerium* in comparison with the 1-year ages. *Strelitziana* was found to be the dominant genus in plant biomass in muscadine grape skins (Sun et al., 2021) and *Pinus massoniana* (Chen et al., 2023), whereas *Trichomerium* was low in abundance in muscadine grape skins but played an important role in *Pinus massoniana*. Further investigation is needed to understand their effect on the host.

Recent evidence suggests that stress and defense response signaling play a role in the production of plant metabolites (SMs) (Isah, 2019). Fungal endophytes, as a phylogenetically and functionally diverse group, can interact with plants in various ways (Chen et al., 2022). Plants adjust their metabolism to environmental conditions, in part through the recognition of a wide array of self and non-self molecules. Among them, endophytes are a provider of the non-self molecules (Bjornson et al., 2021). Differences in endophytic fungal species and abundance must necessarily induce a direct response to host transcription. The transcriptional landscape of *D. nobile* is significantly different along the ages axis with distinct endophytes, but the analysis of the transcriptional landscape is complicated due to the numerous influencing factors. An interesting syndrome came to light through the differential expression analysis of ASV as well as the module analysis of WGCNA. The following genes specificity was summarized in mediumpurple3 module and varied significantly among samples, and correlated with plant hormone metabolism. Plant hormones are a key signaling mechanism for plants to respond to external abiotic factors, and then regulate plant morphology, growth, and synthesis of secondary metabolites (Blazquez et al., 2020). They can also mediate the interaction between plants and microorganisms, either positively or negatively (Kou et al., 2021). In our study, we found that some genes involved in plant hormone signaling, such as NPR1, SAUR, AUX/IAA and ARF, were differentially expressed among different ages and correlated with endophytic fungi and dendrobine content. NPR1 (gene-KFK09_020743) is a key node in signaling downstream from Salicylic acid (SA). It activates defense gene transcription under pathogen challenge (An and Mou, 2011). Auxin plays a crucial role in the process from embryogenesis to senescence in plants, much of which is achieved through the regulation of auxin response genes. As the largest family of early auxin response genes, gene KFK09_019829 (small auxin upregulated RNA (SAUR)) plays an key role in plant growth and development (Li et al., 2021). Auxin-responsive protein IAA (AUX/IAA) Aux/IAA proteins are short-lived nuclear proteins comprising several highly conserved domains that are encoded by the auxin early response gene family (Luo et al., 2018). We designed primers for these four key genes for RT-PCR and obtained the same trend of changes as RNA-seq (Supplementary Figure S4). Further elaboration, they all are the key regulatory nodes of different plant hormones.

Alkaloids, a diverse group of nitrogen-containing secondary metabolites, exhibit a range of biological activities and pharmacological effects (Bhambhani et al., 2021). Among these alkaloids, dendrobine plays a crucial role in *Dendrobium nobile*, displaying antioxidant properties (Hsu et al., 2022) and hepatoprotective effects (Zhou et al., 2020). Despite its significance, the biosynthesis of dendrobine remains poorly understood, although it is hypothesized to involve two major pathways: the mevalonate (MVA) pathway and the methylerythritol phosphate (MEP) pathway (Gong et al., 2021). The MVA pathway generates isopentenyl diphosphate (IPP) and dimethylallyl diphosphate (DMAPP), which serve as precursors for terpenoids. On the other hand, the MEP pathway produces 1-deoxy-D-xylulose-5-phosphate (DXP), a precursor for plastidial terpenoids. Both pathways converge at geranyl diphosphate (GPP), which is subsequently converted to dendrobine by terpene synthases (TPS) and other enzymes. In our study, we observed differential expression of genes involved in alkaloid biosynthesis pathways, such as HMGR, PMK, IDI and TPS, were differentially expressed among different ages and correlated with endophytic fungi and dendrobine content. HMGR, a key enzyme in the MVA pathway, catalyzes the conversion of 3-hydroxy-3-methylglutaryl-CoA (HMG-CoA) to mevalonate (MVA). PMK, an enzyme in the MVA pathway, catalyzes the phosphorylation of mevalonate to mevalonate-5-phosphate (MVP). IDI is an enzyme that catalyzes the interconversion of IPP and DMAPP. TPS is a family of enzymes that catalyze the formation of various terpenoids from GPP or other prenyl diphosphates. The differential expression of these genes suggests that *D. nobile* exhibits varying capacities for alkaloid biosynthesis at different ages, influenced by the composition and abundance of the endophytic fungal community.

## 5 Conclusion

Our study provides novel insights into the interaction between endophytic fungi and the host plant, *D. nobile,* along the age gradient. Our results suggest that endophytic fungi may influence the transcriptome of the stem and the synthesis of dendrobine in *D. nobile* by modulating plant hormone signaling and alkaloid biosynthesis pathways. Additionally, we have identified specific fungal genera and gene modules that are associated with dendrobine content. These findings have potential implications for enhancing the quality and yield of *D. nobile* through targeted manipulation of the endophytic fungal community or host plant gene expression. However, it is important to acknowledge the limitations and uncertainties of our study, which should be addressed in future research. Firstly, our sampling was limited to the stems of *D. nobile* at three different ages, which may not fully represent the entire life cycle of the plant, although these ages are typically harvested within 3 years. It would be valuable to expand the sampling to include a broader range of ages and other tissues, such as roots and flowers, to obtain a more comprehensive understanding of the dynamics of the endophytic fungal community and transcriptome in *D. nobile*. Secondly, we solely employed ITS sequencing to identify the endophytic fungi in the stems of *D. nobile*, which may not capture the complete diversity and functionality of the fungal community. It would be beneficial to incorporate other molecular markers or metagenomic approaches to unveil the functional genes and metabolic pathways of the endophytic fungi in *D. nobile*.

## Data availability statement

The data presented in the study are deposited in the NCBI repository, BioProject ID: PRJNA1040334, SRA: SRR26830466, https://submit.ncbi.nlm.nih.gov/subs/bioproject/SUB13972852/overview.

## Author contributions

YZ: Data curation, Writing – original draft. XJ: Validation, Writing – original draft. XL: Validation, Writing – original draft. LQ: Data curation, Writing – review & editing. DT: Methodology, Writing – review & editing. DW: Validation, Writing – review & editing. CB: Resources, Writing – review & editing. JY: Resources, Writing – review & editing. JX: Supervision, Writing – review & editing. YH: Conceptualization, Funding acquisition, Visualization, Writing – review & editing.

## Footnotes

^1^Version 1.2.11, http://ccb.jhu.edu/software/FLASH/

^2^ http://www.genome.jp/kegg/
