# Supplementary figures and images for: Age-dependent dendrobine biosynthesis in Dendrobium nobile: insights into endophytic fungal interactions
Source: Front Microbiol. 2023 Dec 8;14:1294402. doi: 10.3389/fmicb.2023.1294402 (PMC10749937; doi:10.3389/fmicb.2023.1294402)

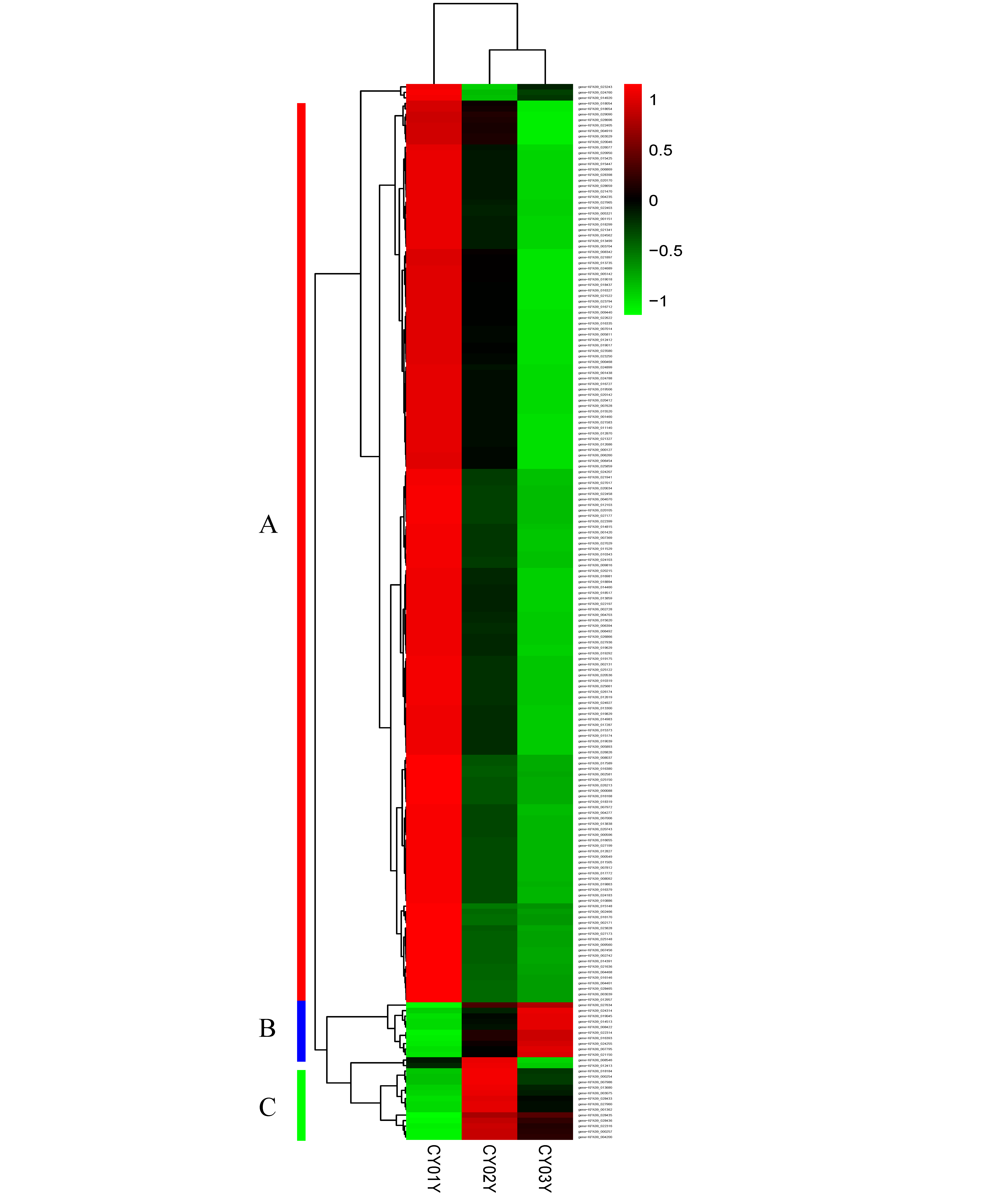

Supplement: Supplementary file 6 [file Image_1.TIF]

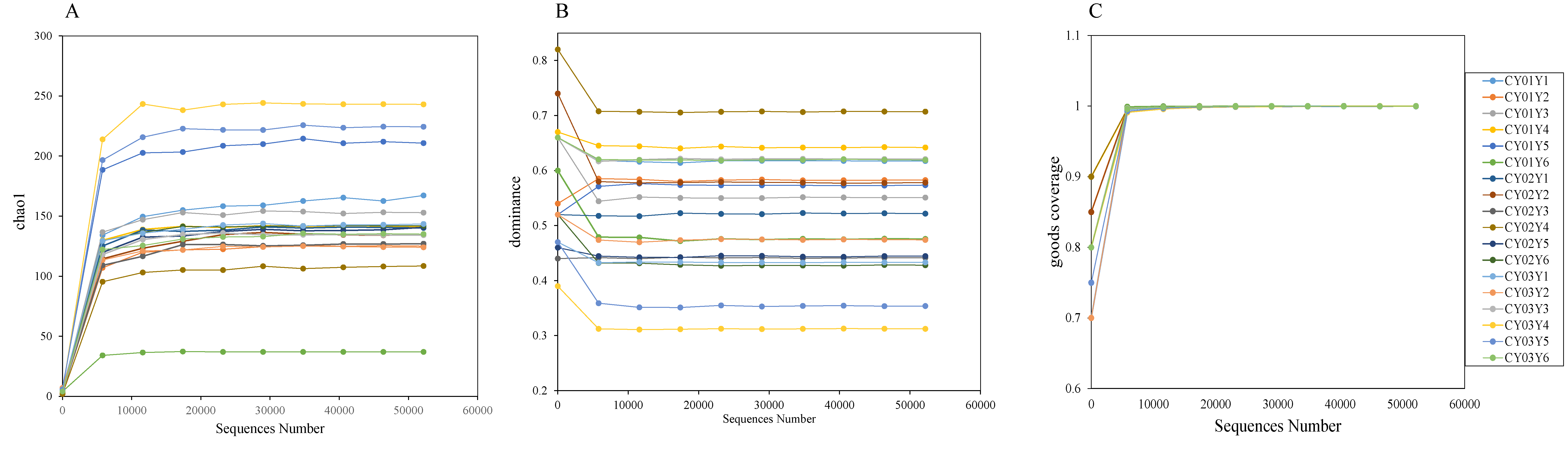

Supplement: Supplementary file 7 [file Image_2.TIF]

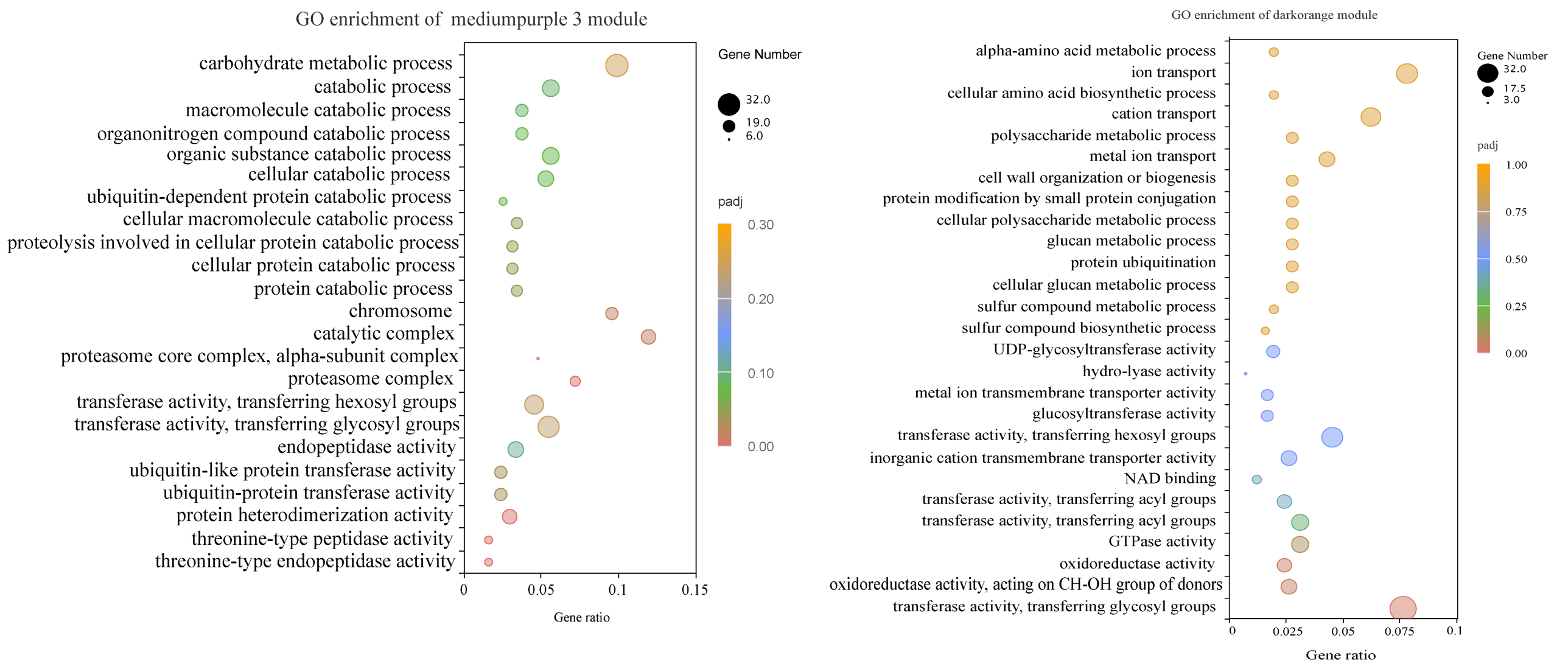

Supplement: Supplementary file 8 [file Image_3.TIF]

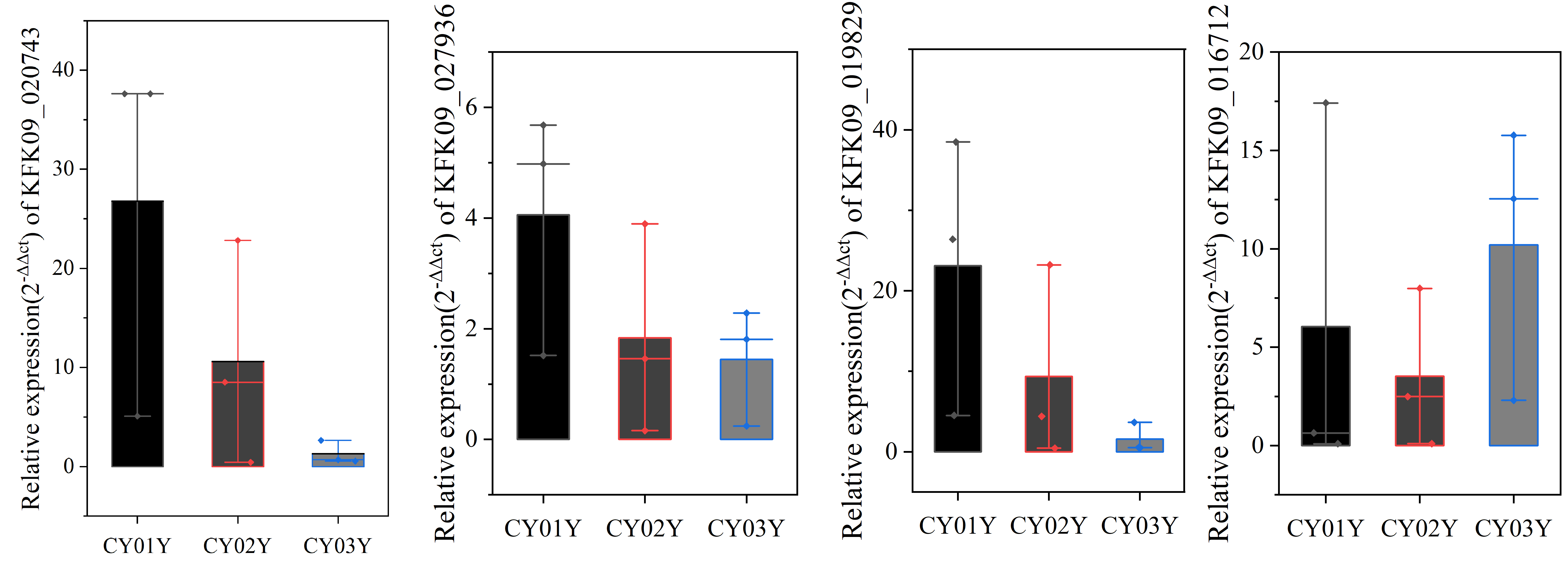

Supplement: Supplementary file 9 [file Image_4.TIF]
